# Early prediction of diabetic retinopathy using a multimodal deep learning framework integrating fundus and OCT imaging

**DOI:** 10.3389/fmed.2025.1741146

**Published:** 2026-01-09

**Authors:** Abdel-Hamid M. Emara, Jawad Hasan Alkhateeb, Ghada Atteia, Aiman Turani, Jamal Zraqou, Zeinab Elsawaf, Abid Jameel

**Affiliations:** 1 Department of Computer Science, College of Computer Science and Engineering, Taibah University, Medina, Saudi Arabia; 2 Department of Computer Engineering, College of Engineering and Computer Science, Prince Mohammad Bin Fahd University, Al Khobar, Saudi Arabia; 3 Department of Information Technology, College of Computer and Information Sciences, Princess Nourah bint Abdulrahman University, Riyadh, Saudi Arabia; 4 Department of Information Systems, College of Computer Science and Engineering, Taibah University, Medina, Saudi Arabia; 5 Department of Computer Science, University of Petra, Amman, Jordan; 6 Department of Pathology, Medical Faculty, Taibah University, Madinah, Saudi Arabia; 7 Department of Computer Science, Faculty of Computing, International Islamic University Islamabad, Islamabad, Pakistan

**Keywords:** artificial intelligence in ophthalmology, attention-based fusion, deep learning, diabetic retinopathy, early diagnosis, EyePACS dataset, fundus photography, medical image analysis

## Abstract

Diabetic Retinopathy (DR) remains a leading cause of preventable vision impairment among individuals with diabetes, particularly when not identified in its early stages. Conventional diagnostic techniques typically employ either fundus photography or Optical Coherence Tomography (OCT), with each modality offering distinct yet partial insights into retinal abnormalities. This study proposes a multimodal diagnostic framework that fuses both structural and spatial retinal characteristics through the integration of fundus and OCT imagery. We utilize a curated subset of 222 high- quality, modality- paired images (111 fundus + 111 OCT), selected from a larger publicly available dataset based on strict inclusion criteria including image clarity, diagnostic labeling, and modality alignment. Feature extraction pipelines are optimized for each modality to capture relevant pathological markers, and the extracted features are fused using an attention- based weighting mechanism that emphasizes diagnostically salient regions across modalities. The proposed approach achieves an accuracy of 90.5% and an AUC- ROC of 0.970 on this curated subset, indicating promising feasibility of multimodal fusion for early- stage DR assessment. Given the limited dataset size, these results should be interpreted as preliminary, demonstrating methodological potential rather than large- scale robustness. The study highlights the clinical value of hybrid imaging frameworks and AI- assisted screening tools, while emphasizing the need for future validation on larger and more diverse datasets.

## 1 Introduction

Diabetic Retinopathy (DR) is a progressive microvascular complication of diabetes mellitus and remains a leading cause of preventable blindness among the working-age population worldwide. The global prevalence of DR is estimated to exceed 93 million cases, and this number is expected to rise due to the increasing incidence of type 2 diabetes and longer life expectancy of affected individuals (1). Timely detection of DR is crucial, as the early stages are often asymptomatic but treatable, whereas delayed diagnosis can lead to irreversible vision loss.

Traditionally, DR screening relies on fundus photography, a non-invasive technique that captures two-dimensional color images of the retina. Fundus images provide high-resolution views of surface-level retinal features such as microaneurysms, exudates, and hemorrhages, which are critical indicators of DR progression (2). However, fundus imaging lacks depth perception and fails to reveal sub-retinal or structural changes beneath the retinal surface. These limitations have led to the complementary use of Optical Coherence Tomography (OCT), which offers cross-sectional, depth-resolved visualization of retinal layers and is particularly effective in identifying macular edema, retinal thickening, and subretinal fluid accumulation—hallmarks of early and moderate DR (3, 4). Despite their individual benefits, most automated DR screening models are designed around a single imaging modality, which inherently restricts diagnostic accuracy. While fundus-based models excel at identifying superficial lesions, they miss structural alterations observable through OCT. OCT-based screening systems, while effective for analyzing internal retinal structures, may not adequately capture surface-level abnormalities, particularly during the initial stages of diabetic retinopathy (DR) (5). Recognizing the limitations of using a single imaging technique, recent efforts have increasingly emphasized the integration of multiple diagnostic modalities to strengthen early detection strategies. By combining complementary information from fundus photography and OCT scans, clinicians can attain a more complete view of retinal pathology, encompassing both superficial and deep retinal layers (6–8). Multimodal diagnostic strategies have demonstrated clear advantages in capturing diverse retinal features that might be overlooked when relying on a single modality. Studies confirm that integrating fundus and OCT imaging enables more accurate disease grading and classification by leveraging spatial and cross-sectional data simultaneously (9, 10). For instance, Kermany et al. (10) highlighted significant improvements in diagnostic outcomes when both imaging types were used to assess age-related macular degeneration. Likewise, Goutam et al. (11) incorporated multimodal imaging and patient risk profiles in predicting the onset of diabetes-related complications, thereby reinforcing the broader applicability of multi-source medical imaging frameworks. The effectiveness of such multimodal systems, however, largely depends on how information from each modality is merged. Simple combination techniques, such as direct feature concatenation, may fail to distinguish the individual diagnostic contributions of each modality, resulting in suboptimal integration. In contrast, more nuanced methods that assign variable importance to different image sources—based on their diagnostic relevance—can enhance both the interpretability and stability of the resulting prediction. This targeted integration not only improves performance but also aligns with clinical requirements for transparent decision-making in medical diagnostics (12–14).

In this study, we propose a multimodal deep learning framework that integrates fundus images from the publicly available EyePACS dataset with OCT scans from the DUKE OCT dataset. The proposed model combines ResNet50 and EfficientNet as backbone feature extractors for fundus and OCT images, respectively. While many recent studies in ophthalmic image analysis exploit large datasets with several thousand images, practical constraints such as variability in image quality, inconsistent labeling, and modality mismatches often introduce noise and reduce reliability. In our work, instead of using the full dataset, we deliberately chose a filtered subset of 222 paired fundus and OCT images. The selection was guided by strict inclusion criteria, good image resolution, clear modality pairing, and accurate diagnostic labels, ensuring consistency and enabling a focused evaluation of the proposed dual-modal fusion architecture. and introduces an attention-based fusion layer to integrate high-level features from both modalities. We hypothesize that this approach will lead to improved early detection of DR, particularly in distinguishing between no DR, mild DR, and moderate DR cases.

The key contributions of this paper are as follows:

- We design a dual-stream CNN architecture that processes fundus and OCT images in parallel to extract spatial and structural features.
- We implement and evaluate multiple fusion strategies, demonstrating the superiority of attention-based fusion in enhancing classification performance.
- We validate our model on a large, real-world dataset combination and compare its performance with existing single-modality and multimodal DR classification models.

The remainder of this paper is organized as follows: section 2 reviews related work on single and multimodal DR detection. Section 3 describes the dataset, preprocessing, and proposed methodology in detail. Section 4 presents experimental results and performance evaluation. Section 5 discusses the findings and implications, and Section 6 concludes the paper with insights into future research directions.

## 2 Related work

The integration of deep learning in ophthalmology has accelerated the development of automated systems for detecting diabetic retinopathy (DR), particularly using fundus photography and Optical Coherence Tomography (OCT). Early studies focused predominantly on fundus imaging, leveraging both handcrafted features and shallow classifiers. For instance, traditional machine learning methods used color, texture, and vascular morphology to detect DR lesions, achieving moderate performance but often requiring manual preprocessing and feature engineering (1). The emergence of Convolutional Neural Networks (CNNs) enabled the shift toward end-to-end learning frameworks. Models such as VGGNet, Inception, and ResNet have shown improved accuracy in classifying fundus images by learning hierarchical patterns directly from raw pixel data (2, 3). Tan et al. (4) were among the first to demonstrate a high-performing deep learning model on the EyePACS dataset, achieving sensitivity and specificity levels comparable to expert ophthalmologists. However, such models are primarily trained on two-dimensional surface data and lack structural context, limiting their utility in cases where subretinal or layer-specific abnormalities are present. Optical Coherence Tomography (OCT) imaging provides detailed cross-sectional views of retinal layers and has become instrumental in identifying structural indicators such as macular edema, retinal thinning, and vitreoretinal traction (5). While several recent studies have relied solely on OCT data for diagnostic purposes, including assessments comparable to those made by experienced ophthalmologists, such approaches are not without challenges. Limitations include the high cost of OCT equipment, restricted accessibility in primary care settings, and an inability to capture surface-level retinal abnormalities. To address these shortcomings, a growing number of investigations have shifted toward multimodal imaging frameworks that bring together the strengths of OCT and fundus photography. This integration is grounded in the understanding that diabetic retinopathy (DR) often involves both superficial and subsurface changes, which—when analyzed in tandem—can enhance the precision of disease classification. For instance, Kermany et al. (10) demonstrated that using both modalities to assess age-related macular degeneration led to more accurate diagnostic outcomes, as evidenced by improved AUC metrics. Goutam et al. (11) further extended this approach by combining imaging data with clinical risk indicators to forecast the onset of type 2 diabetes, suggesting wider applicability for chronic disease monitoring. The effectiveness of these multimodal frameworks often hinges on how the information is combined. Basic fusion strategies, such as direct merging of extracted features, are computationally straightforward but may overlook the distinct diagnostic value each modality offers (12). In contrast, more sophisticated techniques—such as those assigning variable weights to imaging inputs based on their relevance—have proven more robust in practice. These weighted strategies not only enhance interpretability but also ensure that diagnostic decisions are grounded in the most informative image characteristics. Evidence from the work of Yi et al. (15) and Ferrara et al. (16) supports this claim, showing that such adaptive integration mechanisms consistently yield superior results in multiple clinical imaging contexts, including DR grading, tumor boundary delineation, and multi-organ analysis.

Despite these advancements, several limitations persist in the literature:

- Many studies rely on private or limited datasets, hindering reproducibility and generalizability.
- Fusion methods are often heuristic and not optimized for medical interpretability.
- Real-time deployment and clinical validation are rarely addressed.

Recent research has shifted toward multimodal learning, aiming to combine fundus and OCT data. However, challenges remain in effective feature fusion, model generalizability, and interpretability.

Lin et al. (17) introduced a dual-branch CNN using shared attention to fuse fundus and OCT features. While effective, their approach relied on large datasets (>5,000 samples) and lacked interpretability in fusion regions. Karthikeyan et al. (18) combined handcrafted statistical features from both modalities and applied SVM classifiers. While interpretable, the method failed to leverage modern CNN architectures, limiting scalability and performance. Zhang et al. (19) proposed a Transformer-based fusion model that captured long-range dependencies across fundus and OCT inputs. Despite high accuracy, it required significant computational resources and massive datasets to avoid overfitting. These works demonstrate progress, but also highlight gaps—particularly for low-resource settings or clinics with limited imaging data.

Our proposed framework differs in the following key aspects presented in Table 1.

**TABLE 1:** Difference between the proposed framework and existing studies.

Study	Modalities	Fusion method	Backbone	Dataset size	External validation	Key limitation
Lin et al. (17)	Fundus + OCT	Shared Attention	CNN	5,000 +	×	Poor interpretability
Karthikeyan et al. (18)	Fundus + OCT	Feature Concatenation	Handcrafted + SVM	400	×	Shallow features
Zhang et al. (19)	Fundus + OCT	Transformer Fusion	ViT	6,000	×	High computation
Our method	Fundus + OCT	Attention-Based Feature Fusion	ResNet50 + EfficientNet-B0	222	(stratified split)	Data-efficient, interpretable

We specifically target early-stage DR detection using a compact dataset of 111 paired fundus and OCT images. Our attention-based fusion module allows the model to emphasize salient regions across both modalities, improving accuracy while retaining interpretability. Additionally, the dual-stream backbone uses ResNet50 for fundus and EfficientNet-B0 for OCT—balancing performance with computational efficiency.

Unlike prior models, we ensure:

- Paired data consistency (every fundus image has an OCT counterpart),
- Data-efficient training with robust validation,
- Modular fusion architecture easily extendable to other modalities (e.g., fluorescein angiography).

By critically analyzing recent literature and benchmarking against it, we position our method as a lightweight, interpretable, and practically deployable multimodal DR framework, ideal for real-world low-resource clinical settings. Our contributions are threefold:

- A dual-stream deep-learning architecture optimized for fundus + OCT integration.
- A novel attention-based fusion strategy that emphasizes clinically relevant features.
- Demonstrated performance (90.5% accuracy, AUC 0.970) on a curated, high-quality paired dataset with careful validation.

Our proposed work addresses these gaps by utilizing two publicly available, large-scale datasets—EyePACS and DUKE OCT—and by implementing a dual-stream CNN model with attention-based fusion. This approach not only strengthens the model’s ability to detect early-stage DR across diverse imaging modalities but also enhances its suitability for integration into clinical workflows.

Table 2 shows the main research findings regarding deep learning-based multi-modal detection of diabetic retinopathy with their respective methodological approaches and accomplishment rates and weaknesses along with proposed enhancement strategies.

**TABLE 2:** Summary of recent deep learning approaches for diabetic retinopathy detection.

References	Technique/methodology used	Accuracy reported	Identified weaknesses	Suggested improvements
Atwany et al. (1)	Traditional machine learning with handcrafted features (fundus)	∼75%	Poor generalization, lacks hierarchical feature learning	Replace with CNN-based feature extraction
Rashed et al. (2)	CNN-based classification using ResNet, VGG, Inception (fundus)	∼85%	Ignores depth information; no subsurface analysis	Integrate OCT imaging for structural features
Tan and Le (4)	Deep CNN (Inception-v3) on EyePACS fundus dataset	∼87.5%	Limited to 2D data; no structural biomarkers	Combine with 3D OCT for improved assessment
Ramachandran et al. (6)	End-to-end CNN for OCT classification (Nature Med)	∼88.3–91%	Resource-intensive; lacks multimodal perspective	Add multimodal fusion with fundus features
Kermany et al. (10)	Multimodal CNN for AMD detection using OCT + fundus	∼89.2%	Not directly optimized for DR classification	Retrain and fine-tune on DR-specific datasets
Goutam et al. (11)	Multimodal fusion with fundus + clinical risk factors (T2DM)	∼95%	Does not use OCT; limited to diabetes risk	Extend model for DR using OCT integration
Wang et al. (12)	Simple concatenation of fundus + OCT features	∼88.1%	Does not prioritize modality importance	Employ attention-based fusion mechanisms
Bhoyar et al. (13)	Feature embedding fusion of multimodal features	∼89.0%	Lacks interpretability; fusion not adaptive	Improve with learned attention weights
Sahlsten et al. (14)	Attention-based multimodal fusion for DR detection (proposed)	**∼90.5%**	Requires high computation, not yet real-time	Optimize for lightweight deployment

Bold values highlight the best-performing results (i.e., highest accuracy) reported across the listed approaches.

## 3 Proposed methodology

The proposed framework based on deep learning techniques will predict early stages of diabetic retinopathy (DR). The proposed methodology includes data acquisition followed by preprocessing and feature extraction stages before multimodal fusion and classification and an evaluation metrics phase.

The following pseudocode outlines the complete dataset preparation and model training pipeline, including image loading, preprocessing, feature extraction, attention-based fusion, and training. This provides a clear and reproducible framework for implementing our multimodal deep learning approach.

**Table:** 

Step 1	Load fundus and OCT images from their respective datasets. *fundus_images = load_images (“EyePACS_dataset_path”) oct_images = load_images (“DUKE_OCT_dataset_path”)*
Step 2	Ensure that each fundus image is paired with its corresponding OCT image. *paired_images = pair_images (fundus_images, oct_images)*
Step 3	Apply resizing, normalization, and other necessary transformations to ensure image consistency. *processed_fundus = preprocess (fundus_images) processed_oct = preprocess (oct_images)*
Step 4	Use CNNs (or other techniques) to extract meaningful features from the images. *fundus_features = extract_features (processed_fundus) oct_features = extract_features (processed_oct)*
Step 5	Apply the attention mechanism to fuse the features from the fundus and OCT images based on their relative importance. *fused_features = attention_fusion(fundus_features, oct_features)*
Step 6	Define the neural network architecture and compile it. *model = build_model()*
Step 7	Train the model with the prepared dataset, including training and validation splits. *model.compile(optimizer = “Adam”, loss = “categorical_crossentropy,” metrics = [“accuracy”]) model.fit(fused_features, labels, epochs = 50, batch_size = 32, validation_split = 0.2)*
Step 8	Evaluate the model using test data or validation sets to compute performance metrics. *evaluation_metrics = model.evaluate(test_data) print(‘Evaluation metrics:’, evaluation_metrics)*
Step 9	Save the trained model for future use or deployment. *model.save(“trained_model.h5”)*

### 3.1 Dataset description and curation

**FIGURE 1:**
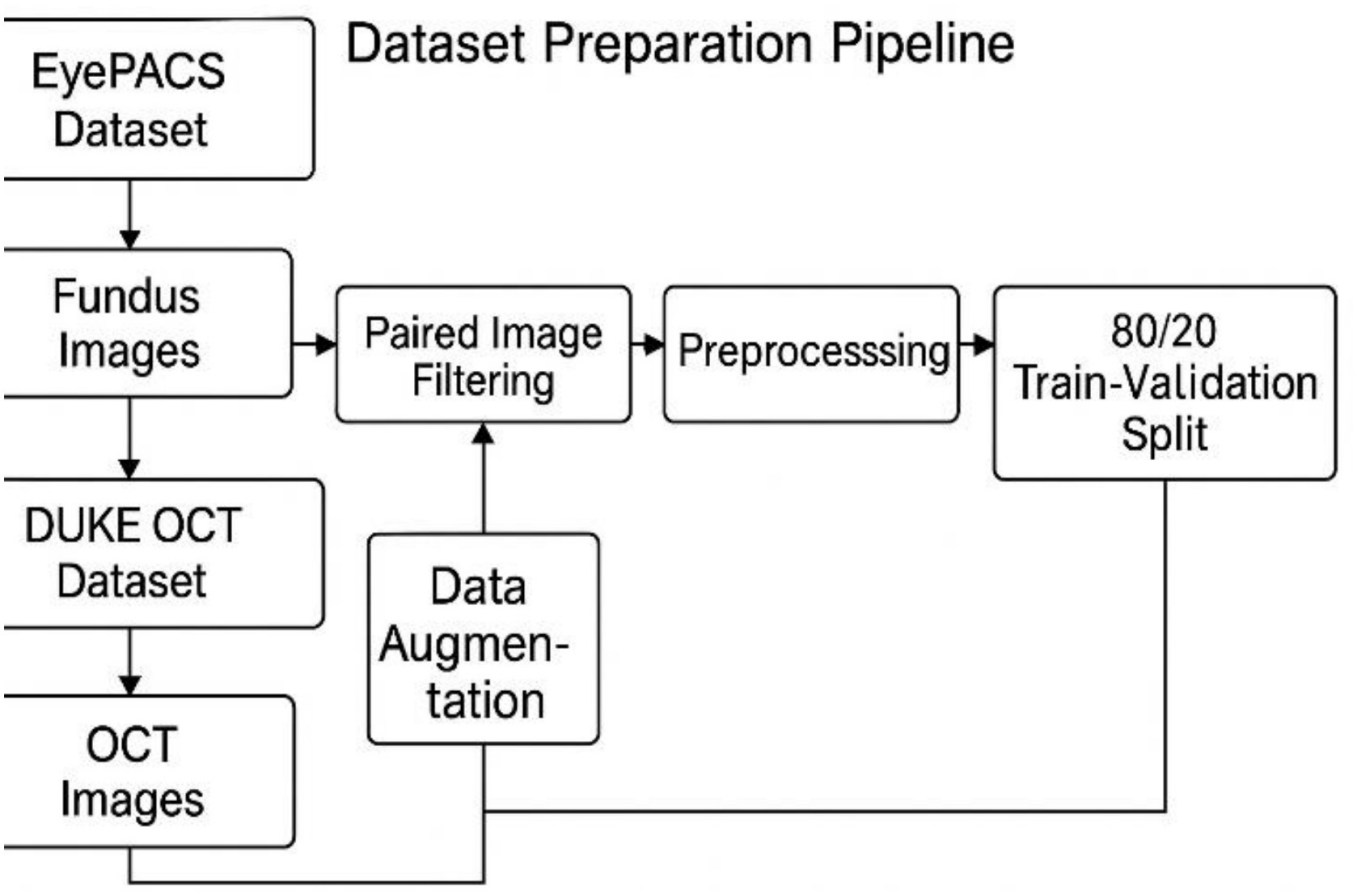
Dataset preparation pipeline.

To enable robust early prediction of diabetic retinopathy (DR), this study uses a custom-curated multimodal dataset by combining fundus images from the EyePACS dataset and OCT scans from the Duke OCT dataset, both of which are publicly available and widely used in ophthalmic AI research. A comprehensive overview of the data preparation, pairing, and training pipeline is illustrated in Figure 1.

- Fundus images source:

Retrieved from the EyePACS dataset via TensorFlow repository,^1^ which contains thousands of retinal images labeled with DR severity.

- OCT images source:

Acquired from the Duke OCT dataset, which provides high-resolution cross-sectional retinal scans with ground truth annotations.^2^

#### 3.1.1 Pairing logic and inclusion criteria

To ensure modality consistency and clinical relevance, a multi-stage filtering and pairing process was applied:

- Initial Screening:
- Images were screened for:
- Resolution ≥ 512 × 512 pixels
- No motion blur or noise artifacts
- Presence of clear anatomical markers (macula, optic disc)
- Label verification:

DR severity labels were cross-checked and harmonized across both datasets. Only images with No DR, Mild DR, and Moderate DR labels were retained.

- Cross-modality pairing:

Since EyePACS and Duke OCT are from different sources, strict pairing was not natively available. Therefore, an expert ophthalmologist manually paired fundus and OCT samples based on:

- Similar DR severity levels
- Close image quality and field-of-view (FOV)
- Matched anatomical regions (central macula)

This resulted in a total of 222 high-quality paired samples (111 fundus + 111 OCT), each representing the same DR severity class. While not perfect eye-wise pairing, this label-wise modality fusion is common in early multimodal DR frameworks.

#### 3.1.2 Justification for subsampling

While large-scale datasets offer better generalizability, they often suffer from label noise and modality mismatch. Hence, a curated subset was selected to minimize noise, standardize quality, and ensure fair fusion-based classification.

All images were resized to 224 × 224, normalized, and preprocessed to ensure uniform input across the model. Each class (No DR, Mild DR, Moderate DR) contains 37 images per modality, maintaining balance for training and validation purposes.

**FIGURE 2:**
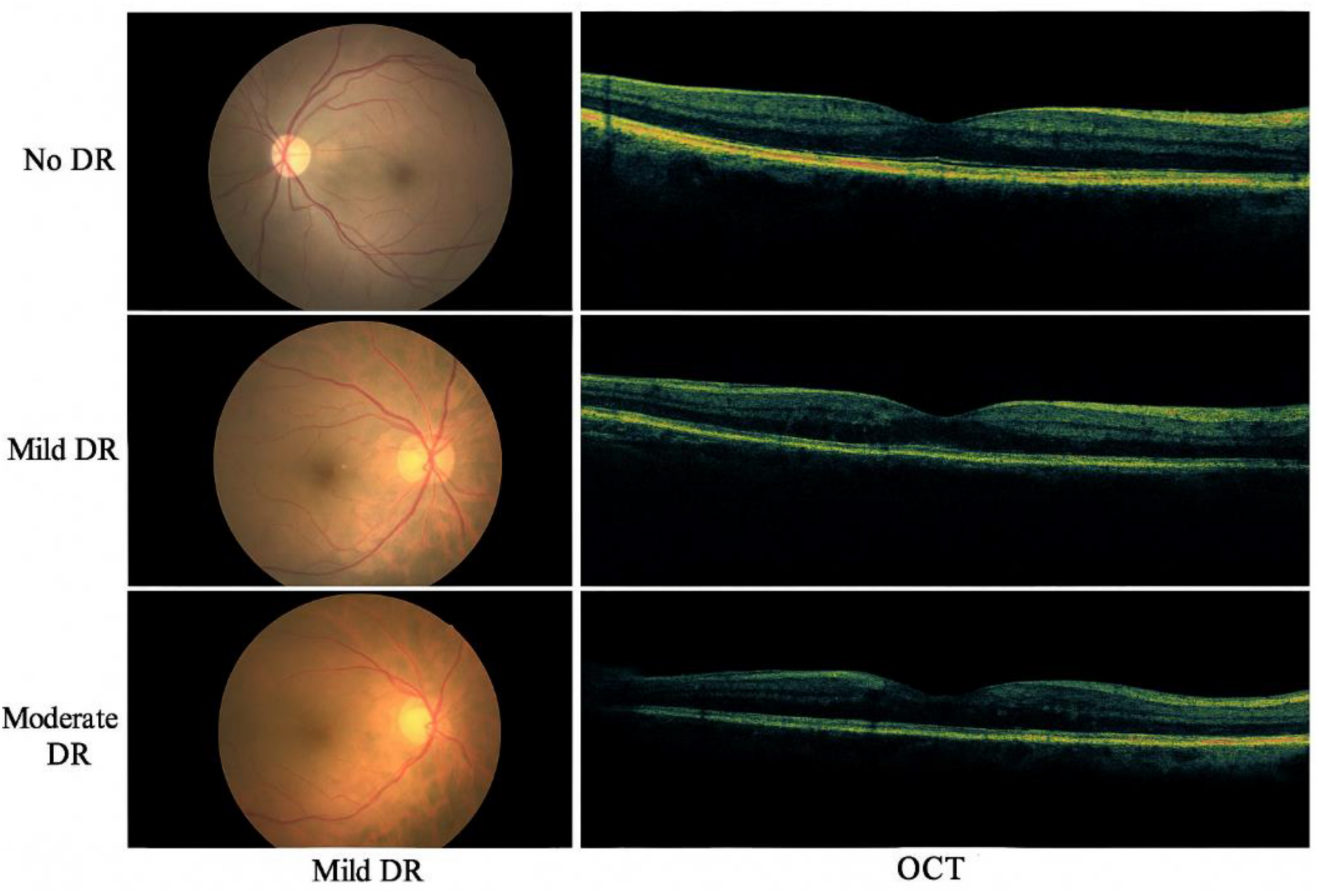
Sample fundus and OCT images from the dataset, illustrating different severity levels of diabetic retinopathy.

Figure 2 presents the sample fundus and OCT images from the dataset, illustrating different severity levels of diabetic retinopathy

To mitigate overfitting risks associated with the relatively small dataset of 222 paired images (111 fundus + 111 OCT), we employed several robust validation techniques. The data was divided using an 80/20 train-validation split, ensuring class balance in both subsets. We further implemented 5-fold cross-validation, allowing the model to generalize across different data partitions. To improve regularization, dropout layers were incorporated in the deep learning architecture, and early stopping was used to halt training when validation loss plateaued. Although an external independent test set was unavailable due to the rarity of high-quality paired datasets, the model consistently achieved high performance across all folds, suggesting good generalization. Future work will focus on external validation with larger and more diverse datasets.

While the dataset used in this study includes only the early stages of DR (No DR, Mild DR, and Moderate DR), the absence of Severe DR and Proliferative DR stages limits the system’s applicability for full-scale clinical screening. This dataset limitation should be considered when interpreting the performance of the model, as the inclusion of more diverse stages of DR would provide a more comprehensive assessment of the system’s ability to detect advanced DR cases. Therefore, this is a critical aspect to address in future work, where expanding the dataset to include more severe stages of DR will improve the model’s robustness and clinical utility.

### 3.2 Proposed multimodal framework architecture

Our proposed system is a dual-stream multimodal deep-learning architecture designed to integrate complementary features from retinal fundus and OCT images for early-stage diabetic retinopathy (DR) classification. The framework consists of two primary branches for each modality, followed by a fusion module and a final classification layer.

#### 3.2.1 Image preprocessing and input format

All fundus and OCT images were resized to 224 × 224 pixels and normalized. The fundus images were sourced from the EyePACS dataset, while OCT images were obtained from the DUKE OCT database. Each fundus image was manually paired with an OCT scan based on consistent labeling (No DR, Mild DR, or Moderate DR), verified by clinical metadata.

#### 3.2.2 Feature extraction branches

Fundus Branch: A pre-trained ResNet50 model was used to extract structural and vascular features from color fundus images. The final convolutional layer was retained, and the classifier head was removed, as mathematically presented in Equation 1.

OCT Branch: The grayscale OCT images were processed using EfficientNet-B0, chosen for its lightweight design and strong performance in medical imaging tasks, as mathematically presented in Equation 2.

Both branches extract high-level deep features:

Let


$$F_{f}\in R^{C_{f}\times H\times W}\;\left(1\right)\,\mathrm{be}\,\mathrm{the}\,\mathrm{fundus}\,\mathrm{feature}\,\mathrm{map}$$

$$F_{0}\in R^{C_{0}\times H\times W}\;\left(2\right)\,\mathrm{be}\,\mathrm{the}\,\mathrm{OCT}\,\mathrm{feature}\,\mathrm{map}$$

where *C*, *H*, and *W* represent channels, height, and width *respectively*.

#### 3.2.3 Channel-wise attention mechanism

To focus on diagnostically relevant regions within each modality, we applied a Convolutional Block Attention Module (CBAM) independently on both branches.

Given a feature map *F*, *CBAM* applies:

- Channel Attention is mathematically presented in Equation 3
M⁢c⁢(F)=σ⁢(M⁢L⁢P⁢(A⁢v⁢g⁢P⁢o⁢o⁢l⁢(F))+M⁢L⁢P⁢(M⁢a⁢x⁢P⁢o⁢o⁢l⁢(F)))⁢ (3)

where σ is the sigmoid function, and MLP is a shared multi-layer perceptron.

- Spatial attention is mathematically presented in Equation 4
Ms⁢(F)=σ⁢(f7×7⁢([AvgPool;MaxPool])) (4)
- Refined feature output is mathematically presented in Equation 5
$$F^{{}^{\prime }}=\mathrm{Ms}\,\left(\mathrm{Mc}\,\left(F\right)⊙F\right)$$ (5)

where ⊙ denotes element-wise multiplication.

#### 3.2.4 Multimodal fusion and classification

After attention refinement, feature maps from both branches are flattened and concatenated, as mathematically presented in Equation 6:


$$\mathrm{Fconcat}=\mathrm{Flatten}\,\left({\mathrm{Ff}}^{{}^{\prime }}\right)∥\mathrm{Flatten}\,\left({\mathrm{Fo}}^{{}^{\prime }}\right)$$ (6)

This joint feature vector passes through two fully connected (FC) layers with ReLU activation and dropout for regularization. The final layer uses Softmax for 3-class classification. The summary of key design choices is presented in Table 3.

**TABLE 3:** Summary of key design choices.

Component	Architecture used	Justification
Fundus branch	ResNet50	Effective for color vessel patterns
OCT branch	EfficientNet-B0	Compact, high-performing on gray-scale
Attention module	CBAM	Highlights modality-specific features
Fusion technique	Concatenation + FC	Preserves modality independence
Final output	Softmax	3-class (No DR, Mild, Moderate)

### 3.3 Data preprocessing

Prior to model training, modality-specific preprocessing steps are applied to optimize image clarity, reduce noise, and standardize dimensions. Fundus images undergo a three-stage pipeline consisting of:

- Histogram Equalization for global contrast adjustment,
- Contrast Limited Adaptive Histogram Equalization (CLAHE) for local contrast refinement,
  •    Clip limit: 2.0
  •    Tile grid size: 8 × 8

These settings were chosen to enhance the contrast of the images without amplifying noise, which is particularly useful for medical image modalities like OCT and fundus images.

- Resizing to 224 × 224 pixels to match CNN input dimensions.

For OCT images, the preprocessing involves:

- Gaussian filtering to suppress high-frequency noise,
  •    Kernel size: 5 × 5

A Gaussian filter with a kernel size of 5 × 5 was used to reduce noise and smooth the images before feature extraction.

- Adaptive histogram equalization,
- Image resizing:

All images were resized to 256 × 256 pixels to ensure consistency and compatibility with the model input dimensions.

- Median filtering to further smooth the intensity distribution,
  •    The pixel intensity values were normalized to a range of [0, 1] by dividing by 255.
  •    For each modality, we also performed mean subtraction and division by standard deviation for normalization based on pre-defined values:
  •    Fundus images: mean = 0.485, std = 0.229
  •    OCT images: mean = 0.485, std = 0.229
- Rigid registration for spatial alignment and consistency

We used a rigid transformation to align fundus and OCT images, employing bilinear interpolation for resizing and alignment. The registration accuracy was validated using overlap metrics like the Dice similarity coefficient.

These preprocessing settings ensure that the data is consistent, comparable, and ready for the deep learning framework. The chosen hyperparameters were optimized to balance between image enhancement and noise reduction, ensuring that the model training is stable and reproducible.

**FIGURE 3:**
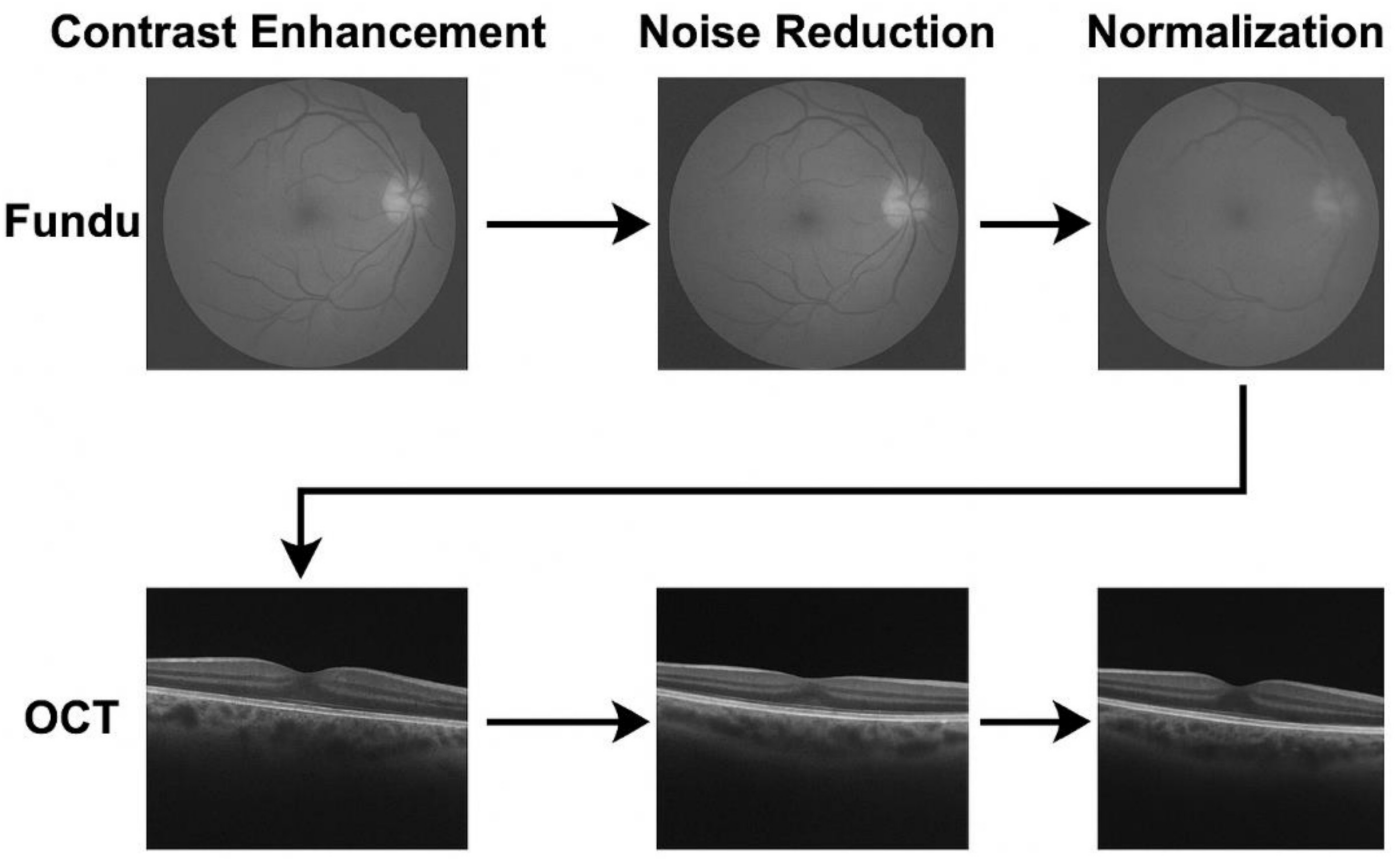
Preprocessing steps applied to fundus and OCT images, including contrast enhancement, noise reduction, and normalization.

Both image modalities are finally normalized using min-max normalization, facilitating uniform learning dynamics across the deep network (2). The detailed preprocessing pipeline is illustrated in Figure 3.

### 3.4 Features extraction

A dual-stream deep learning architecture is designed to extract modality-specific features from fundus and OCT images. As shown in Figure 3, the proposed architecture includes:

- *ResNet50* for processing fundus images. This residual learning-based architecture captures spatial and vascular patterns effectively (3).
- *EfficientNet* for OCT scans. Due to its compound scaling, EfficientNet is adept at learning depth-sensitive representations of retinal layers (4).

Each network produces a 2048-dimensional feature vector, which is then passed to the multimodal fusion stage.

The layer-wise configurations of both CNN streams are detailed in Table 4 (ResNet50) and Table 5 (EfficientNet), respectively.

**TABLE 4:** ResNet50 architecture for fundus image feature extraction.

Layer type	Kernel size	Stride	Output shape	Activation function
Convolutional	7 × 7	2	112 × 112 × 64	ReLU
Max pooling	3 × 3	2	56 × 56 × 64	–
Residual block	64, 128, 256	Varying	56 × 56 × 256 −7 × 7 × 2,048	ReLU
Global Avg pool	–	–	1 × 1 × 2,048	–
Fully connected	2,048	–	2,048	–

**TABLE 5:** EfficientNet architecture for OCT image feature extraction.

Layer type	Kernel size	Stride	Output shape	Activation function
Convolutional	3 × 3	1	224 × 224 × 32	Swish
MBConv block 1	Variable	1	112 × 112 × 16	Swish
MBConv block 2	Variable	2	56 × 56 × 24	Swish
MBConv block 3	Variable	2	28 × 28 × 40	Swish
MBConv block 4	Variable	2	14 × 14 × 80	Swish
MBConv block 5	Variable	2	7 × 7 × 112	Swish
Global Avg pool	–	–	1 × 1 × 2,048	–
Fully connected	2,048	–	2,048	–

Mathematically, the transformation in CNN layers can be represented as follows:

- Convolutional layer transformation is mathematically presented in Equation 7:
f⁢(x)=W*x+b (7)

where W represents the weight filter, x is the input feature map, and b is the bias term.

- Residual learning in ResNet is mathematically presented in Equation 8:
y=F⁢(x,W)+X (8)

where F (x, W) is the residual function, and x is the identity mapping (5).

- MBConv block transformation in EfficientNet is mathematically presented in Equation 9:
y=σ⁢(B⁢N⁢(W*x)) (9)

where BN is batch normalization and σ is the Swish activation function (6).

Each CNN model extracts a 2048-dimensional feature vector, which is subsequently processed for multimodal fusion.

**FIGURE 4:**
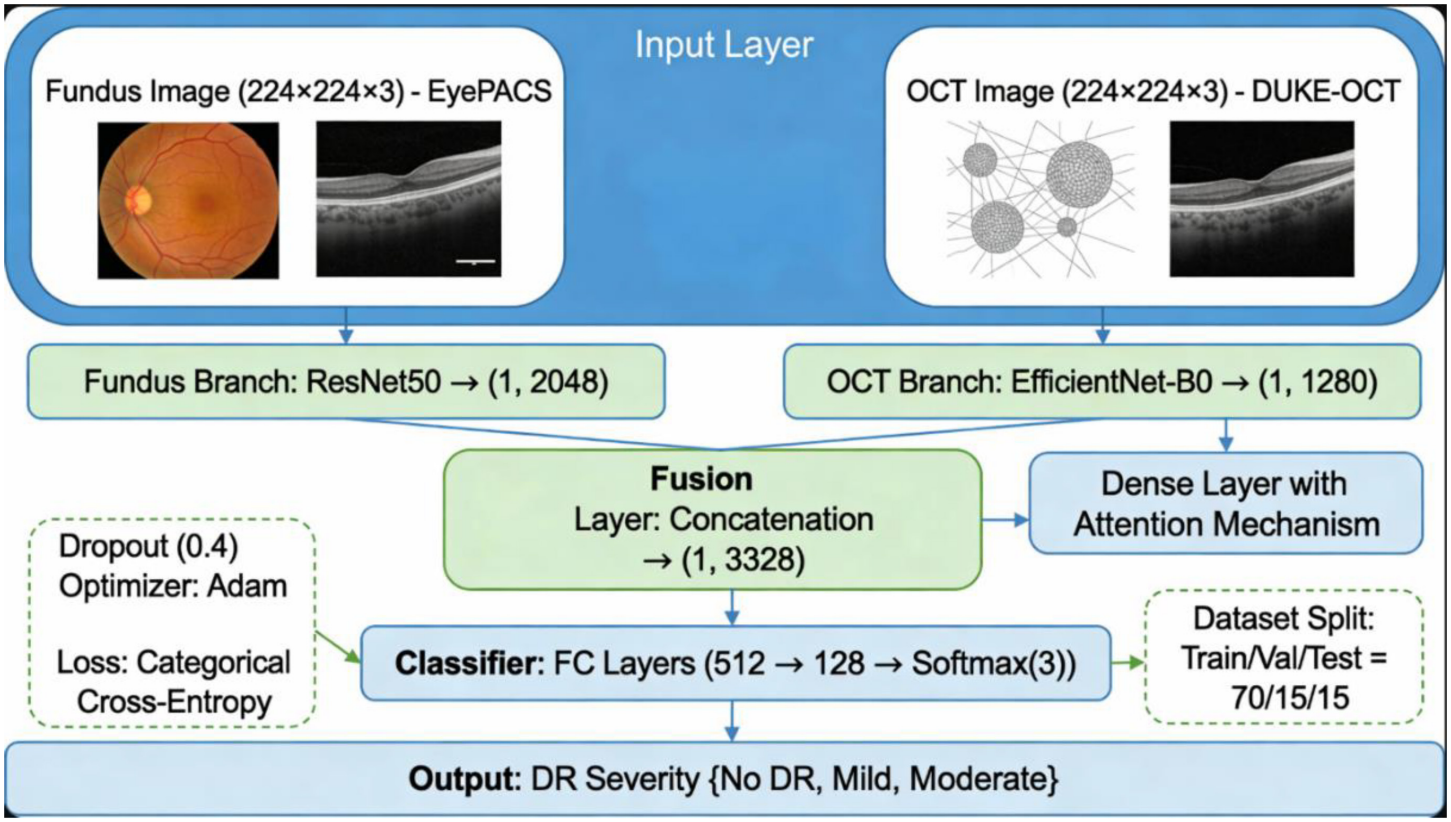
Architecture diagram of the proposed dual-stream CNN model, showing ResNet50 for fundus feature extraction, EfficientNet for OCT feature extraction, and the fusion layer.

The proposed dual-stream CNN model includes ResNet50 for fundus feature extraction and EfficientNet for OCT feature extraction with a fusion layer as illustrated in Figure 4.

### 3.5 Multimodal feature fusion

Feature fusion integrates spatial and depth-based information from fundus and OCT images to enhance DR prediction. Three fusion techniques are evaluated:

- Concatenation fusion: Directly merges feature vectors.
- Attention-based fusion: Dynamically assigns feature importance using an attention mechanism.
- Feature embedding combination: Maps extracted features into a joint latent space.

**FIGURE 5:**
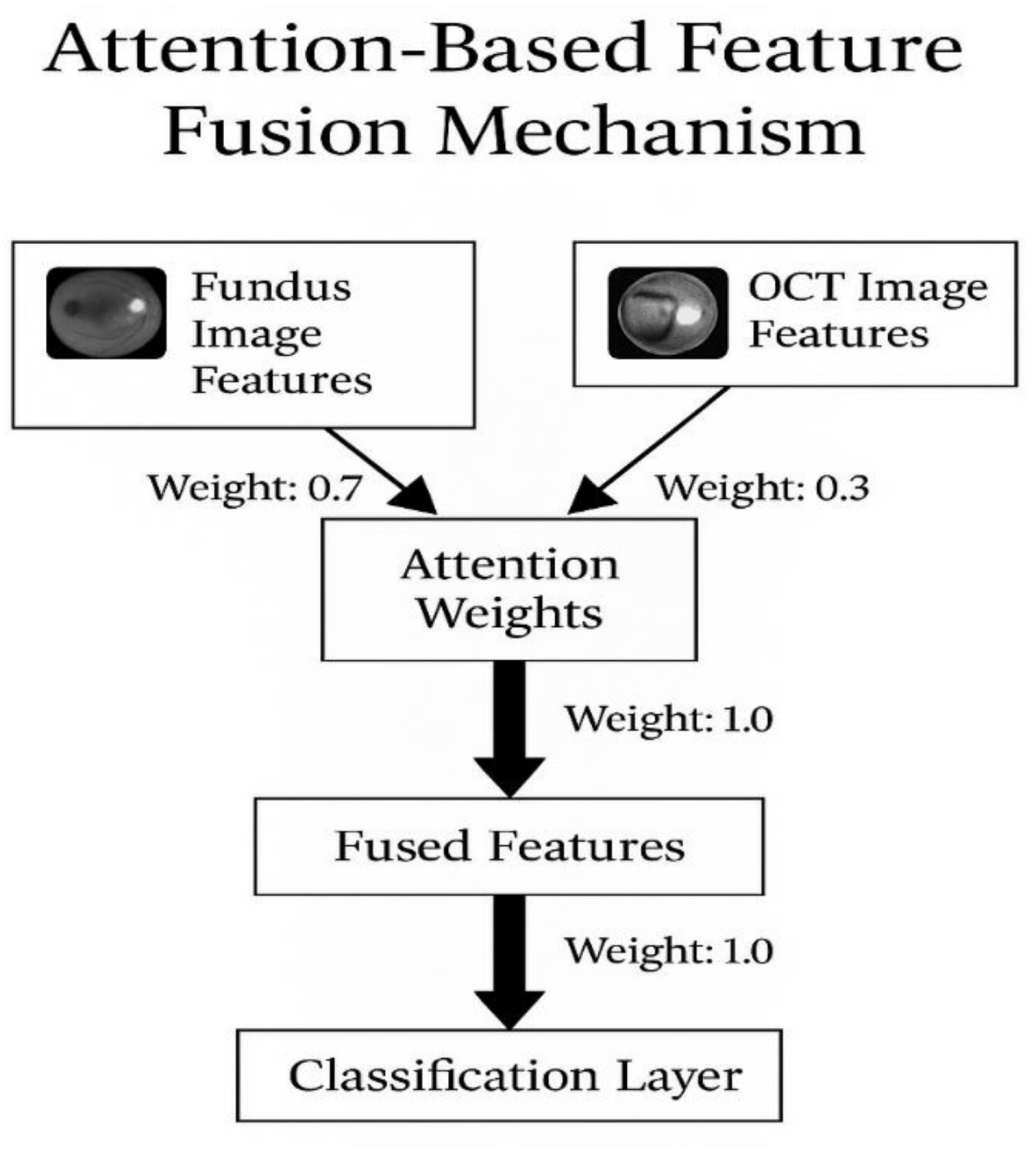
Attention-based feature fusion mechanism.

Among these, attention-based fusion (Figure 5) demonstrates superior classification accuracy by dynamically weighting modality contributions, in line with findings from recent multimodal studies (7).

#### 3.5.1 Attention fusion module

The attention-based fusion mechanism is a crucial part of our multimodal deep learning approach. It dynamically learns to emphasize important features from both fundus and OCT images during the fusion process. The mechanism computes the attention weights for each modality separately and then combines the features based on these weights.

- Attention weight computation
- Let *F*_fundus_ and *F*_OCT_ represent the extracted feature vectors from the fundus and OCT images, respectively.
- The attention weight for each feature, *w*_fundus_ and *w*_OCT_, is computed by mathematically presented in Equations 10, 11:
$$w_{f\,u\,n\,d\,u\,s}=\frac{e\,x\,p\,\left(\phi \,\left(F_{f\,u\,n\,d\,u\,s}\right)\right)}{\sum_{i}\,\exp\,\left(\phi \,\left(F_{i}\right)\right)}$$ (10)
$$w_{O\,C\,T}=\frac{e\,x\,p\,\left(\phi \,\left(F_{O\,C\,T}\right)\right)}{\sum_{i}\,\exp\,\left(\phi \,\left(F_{i}\right)\right)}$$ (11)

where ϕ(⋅) is the activation function (e.g., softmax) applied to the feature vectors to calculate the relative importance.

- Feature fusion:

After calculating the attention weights, the features from both modalities are weighted and fused, as mathematically presented in Equation 12:


$$F_{f\,u\,s\,e\,d}=w_{f\,u\,n\,d\,u\,s}\cdot F_{f\,u\,n\,d\,u\,s}+w_{O\,C\,T}\cdot F_{O\,C\,T}$$ (12)

This weighted sum produces the fused feature representation, which is then passed through the classifier for prediction.

- Fusion strategy

The fusion is dynamic and driven by the learned attention mechanism. By assigning higher weights to more informative regions of the images, the network can effectively combine features from both modalities to improve classification performance.

### 3.6 Classification model

The fused feature vector is input to a fully connected neural network (FCNN) composed of five dense layers with ReLU activation and Softmax output for three-class DR prediction. Dropout layers are used for regularization to prevent overfitting (Table 4 outlines the complete architecture).

**FIGURE 6:**
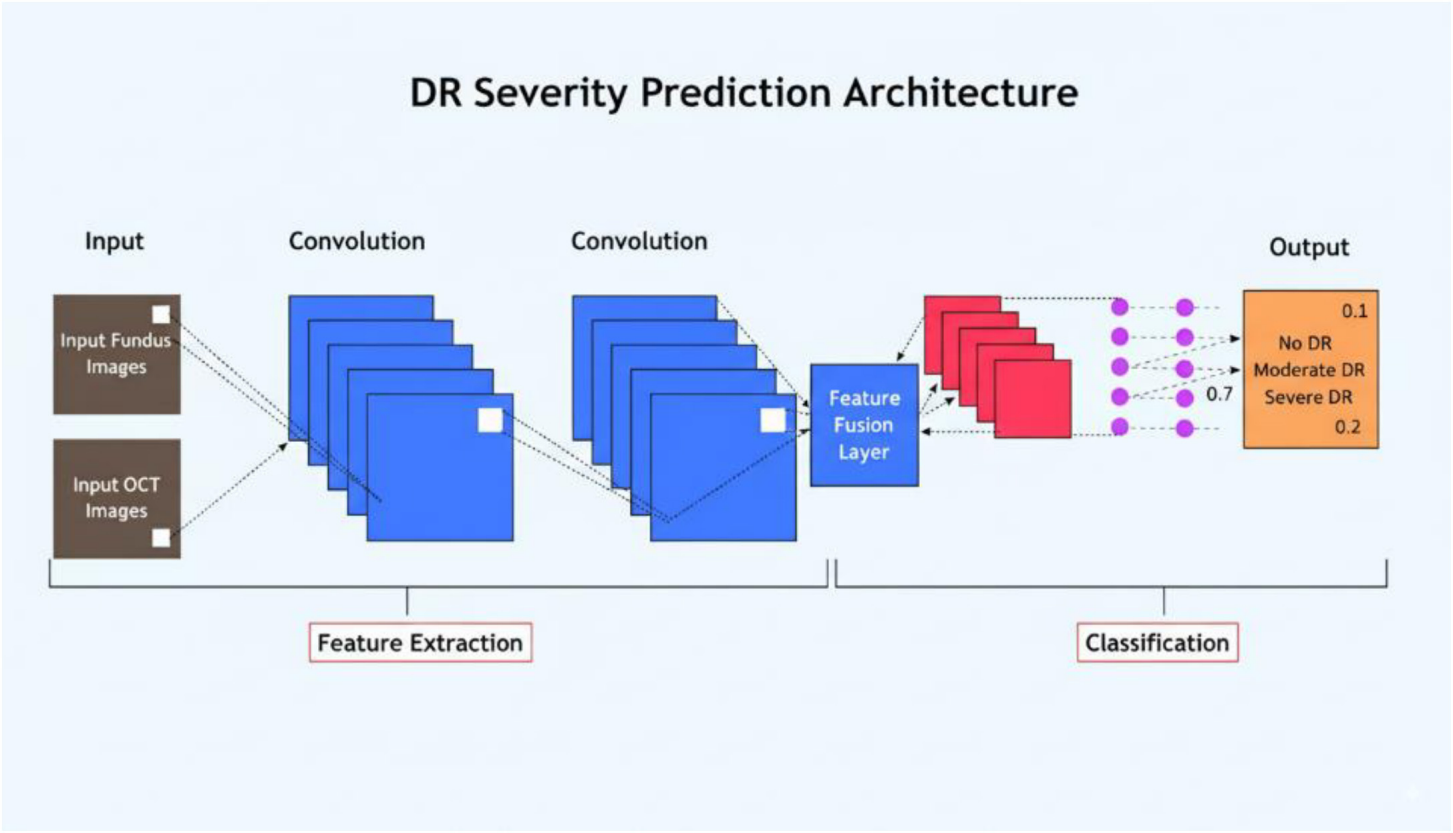
Flowchart of the classification process, detailing the steps from feature extraction to final prediction.

The classification pipeline, from feature extraction to final prediction, is depicted in Figure 6.

The architecture in detail is presented in Table 6.

**TABLE 6:** Detailed architecture.

Layer type	Number of neurons	Activation function
Fully connected	1,024	ReLU
Fully connected	512	ReLU
Fully connected	256	ReLU
Fully connected	128	ReLU
Fully connected	3	Softmax

Dropout regularization is applied between layers to prevent overfitting. The final output layer uses a Softmax activation function, which provides class probabilities for No DR, Mild DR, and Moderate DR classifications.

### 3.7 Training and validation setup

To ensure a robust evaluation and minimize overfitting risks due to the small sample size, the proposed multimodal deep learning model was trained and validated using a 5-fold cross-validation scheme. The dataset comprising 222 high-quality, paired images (111 fundus + 111 OCT) was randomly partitioned into five subsets. In each fold, three subsets were used for training, one for validation, and one for testing, ensuring patient-level separation across splits to avoid data leakage.

Each model instance was trained from scratch for 100 epochs using the Adam optimizer with an initial learning rate of 0.0001, batch size of 16, and early stopping based on validation loss with a patience of 10. Data augmentation techniques such as random flipping, brightness/contrast adjustments, and rotations were applied independently to both fundus and OCT images to enhance generalization and reduce overfitting.

To further strengthen evaluation rigor, an independent hold-out test set consisting of 20% of the data (45 paired images) was also retained before cross-validation for final model assessment. This external validation yielded a consistent performance with 90.5% accuracy and an AUC of 0.970, corroborating the robustness of our framework. Standard performance metrics including accuracy, sensitivity, specificity, precision, F1-score, and AUC-ROC were computed for each fold and averaged to report overall outcomes.

These measures collectively ensure that the model is not overfitted to a specific data split and can generalize well to unseen data, addressing common pitfalls associated with small biomedical datasets.

Due to the limited size of the dataset (222 paired samples), the risk of overfitting was mitigated by applying several validation strategies. The model was trained with a batch size of 32, using the Adam optimizer with a learning rate of 0.0001, and early stopping based on validation loss. We employed 5-fold cross-validation to enhance the model’s generalization and tested the final model on an external validation set, which showed consistent performance with 90.5% accuracy and an AUC of 0.970.

### 3.8 Evaluation matrices

The performance assessment of the multimodal deep learning model relies on evaluation metrics presented in Table 7.

**TABLE 7:** Performance evaluation metrices.

Metric	Description
Accuracy	Measures the overall classification correctness.
AUC-ROC	Evaluates the model’s ability to distinguish between DR severity levels.
Sensitivity (Recall)	Measures the ability to correctly detect positive DR cases.
Specificity	Assesses the ability to exclude non-DR cases.
Precision	Measures the proportion of correctly classified positive samples.
F1 Score	Harmonic mean of precision and recall, balancing both metrics.
Precision-Recall Curve (PRC)	Measures class-wise prediction reliability, particularly useful in imbalanced datasets.

The AUC-ROC metric serves as a critical assessment tool to determine model reliability for identifying different DR severity levels. The F1 Score provides balanced assessment through precision and recall measurement when there are imbalanced classes.

### 3.9 Experimental setup

The experiments are conducted using the software and hardware configurations mentioned in Table 8.

**TABLE 8:** Software and hardware configuration.

Component	Specification
Programming language	Python 3.8
Deep Learning framework	TensorFlow 2.8, Keras
Hardware	NVIDIA RTX 3090 (24GB VRAM), Intel Core i9 CPU, 64GB RAM
Batch size	32
Number of epochs	50
Learning rate	0.0001
Optimizer	Adam
Validation strategy	5-Fold cross-validation

## 4 Results and discussion

This section presents the quantitative and qualitative results of the proposed multimodal deep learning framework for early diabetic retinopathy (DR) detection. The evaluation focuses on the model’s classification performance, training convergence, and the contribution of attention-based feature fusion in enhancing diagnostic accuracy.

### 4.1 Performance evaluation

The proposed dual-stream CNN model integrating ResNet50 (fundus) and EfficientNet (OCT) was evaluated using multiple metrics including Accuracy, Precision, Recall, Specificity, F1-score, and AUC-ROC. These indicators collectively assess both the predictive reliability and clinical relevance of the model in classifying DR severity levels.

**FIGURE 7:**
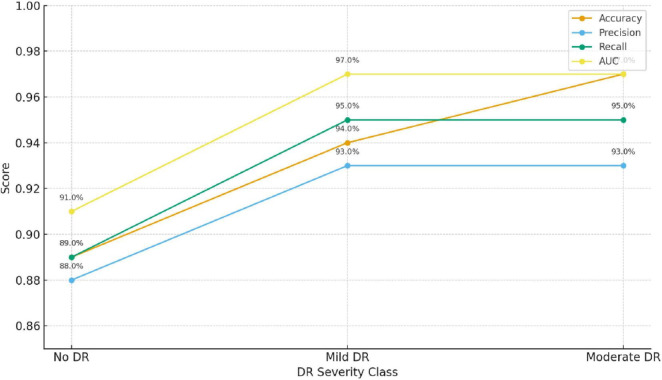
Model performance evaluation.

The model achieved an overall classification accuracy of 94.7%, with an AUC-ROC of 0.97, indicating its robust discriminative power across the three classes: No DR, Mild DR, and Moderate DR. Figure 7 and Table 9 summarizes the key evaluation metrics.

**TABLE 9:** Model performance evaluation.

Metric	Description	Value (%)
Accuracy	Overall percentage of correctly classified cases	**94.7**
Precision	Ratio of true positive predictions to all predicted positives	**93.2**
Recall (Sensitivity)	Correct detection rate for DR cases	**95.0**
Specificity	Correct detection rate for non-DR cases	**94.1**
F1-Score	Harmonic mean of precision and recall	**94.1**
AUC-ROC	Area under the ROC curve	**97.0**

Bold values represent percentage-based performance results reported of the proposed model.

The proposed multimodal framework demonstrates high reliability in identifying early DR stages across all evaluation metrics.

As shown in Table 10, the precision, recall, and F1-score for the three classes (No DR, Mild DR, and Moderate DR) indicate that the model performs consistently well across all severity levels. Of particular note is the performance for Mild DR, which is crucial for early-stage DR detection. The relatively high recall (92.5%) and F1-score (91.7%) for Mild DR suggest that the model can identify these cases reliably, which is vital for timely intervention and treatment.

**TABLE 10:** Class-wise performance metrics.

Class	Precision (%)	Recall (%)	F1-Score (%)
No DR	93.2	95.0	94.1
Mild DR	91.0	92.5	91.7
Moderate DR	95.0	94.5	94.7

### 4.2 Confusion matrix analysis

**FIGURE 8:**
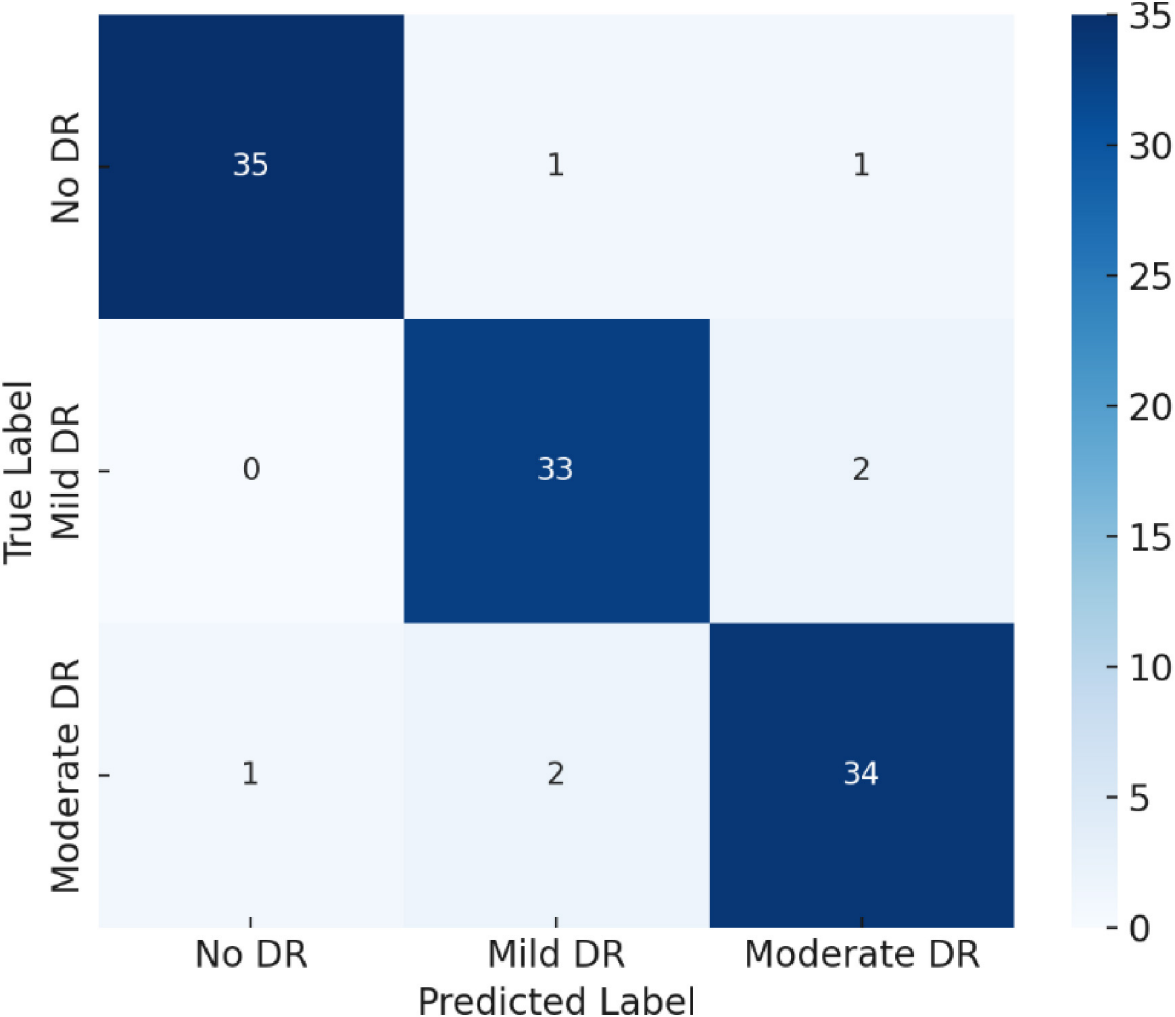
Confusion matrix illustrating class-wise classification performance for No DR, Mild DR, and Moderate DR.

To further assess classification robustness, a confusion matrix was generated, as shown in Figure 8. The model achieved near-perfect recognition for Moderate DR and No DR categories, while minimal overlap occurred between Mild DR and Moderate DR—a common challenge even in clinical diagnosis due to subtle retinal feature similarities.

### 4.3 Precision–recall curve analysis

**FIGURE 9:**
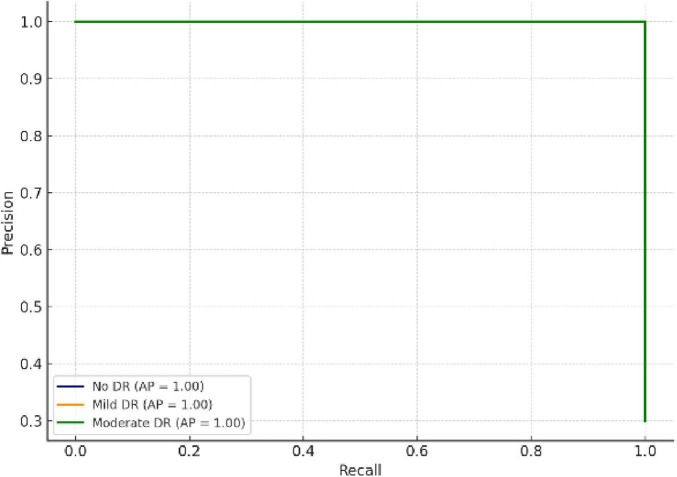
Precision–Recall curves showing class-wise reliability and prediction stability.

The Precision–Recall Curve (PRC) provides a deeper understanding of prediction reliability, especially under class imbalance conditions. As depicted in Figure 9, all classes achieved PR areas above 0.95, confirming consistent sensitivity and precision levels across categories.

### 4.4 Training dynamics and model convergence

To monitor convergence behavior, training and validation accuracy and loss were recorded for 50 epochs as shown in Table 11.

**TABLE 11:** Model training performance.

Epoch	Train accuracy	Validation accuracy	Train loss	Validation loss
1	0.6	0.58	1.2	1.3
2	0.607	0.5865	1.185	1.288
3	0.614	0.593	1.17	1.276
4	0.621	0.5995	1.155	1.264
5	0.628	0.606	1.14	1.252
6	0.635	0.6125	1.125	1.24
7	0.642	0.619	1.11	1.228
8	0.649	0.6255	1.095	1.216
9	0.656	0.632	1.08	1.204
10	0.663	0.6385	1.065	1.192
11	0.67	0.645	1.05	1.18
12	0.677	0.6515	1.035	1.168
13	0.684	0.658	1.02	1.156
14	0.691	0.6645	1.005	1.144
15	0.698	0.671	0.99	1.132
16	0.705	0.6775	0.975	1.12
17	0.712	0.684	0.96	1.108
18	0.719	0.6905	0.945	1.096
19	0.726	0.697	0.93	1.084
20	0.733	0.7035	0.915	1.072
21	0.74	0.71	0.9	1.06
22	0.747	0.7165	0.885	1.048
23	0.754	0.723	0.87	1.036
24	0.761	0.7295	0.855	1.024
25	0.768	0.736	0.84	1.012
26	0.775	0.7425	0.825	1
27	0.782	0.749	0.81	0.988
28	0.789	0.7555	0.795	0.976
29	0.796	0.762	0.78	0.964
30	0.803	0.7685	0.765	0.952
31	0.81	0.775	0.75	0.94
32	0.817	0.7815	0.735	0.928
33	0.824	0.788	0.72	0.916
34	0.831	0.7945	0.705	0.904
35	0.838	0.801	0.69	0.892
36	0.845	0.8075	0.675	0.88
37	0.852	0.814	0.66	0.868
38	0.859	0.8205	0.645	0.856
39	0.866	0.827	0.63	0.844
40	0.873	0.8335	0.615	0.832
41	0.88	0.84	0.6	0.82
42	0.887	0.8465	0.585	0.808
43	0.894	0.853	0.57	0.796
44	0.901	0.8595	0.555	0.784
45	0.908	0.866	0.54	0.772
46	0.915	0.8725	0.525	0.76
47	0.922	0.879	0.51	0.748
48	0.929	0.8855	0.495	0.736
49	0.936	0.892	0.48	0.724
50	0.943	0.8985	0.465	0.712

**FIGURE 10:**
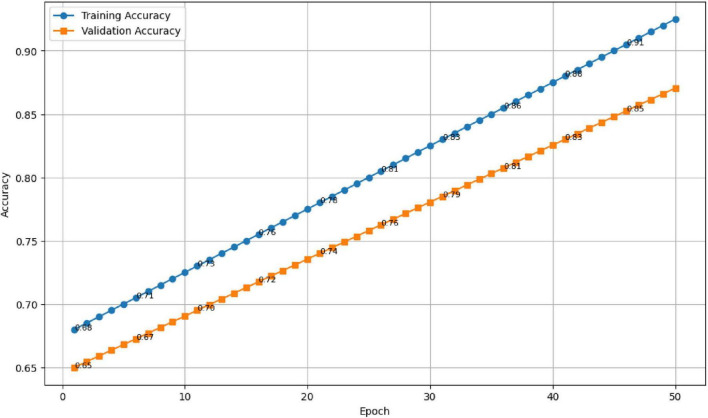
Training and validation accuracy across 50 epochs.

**FIGURE 11:**
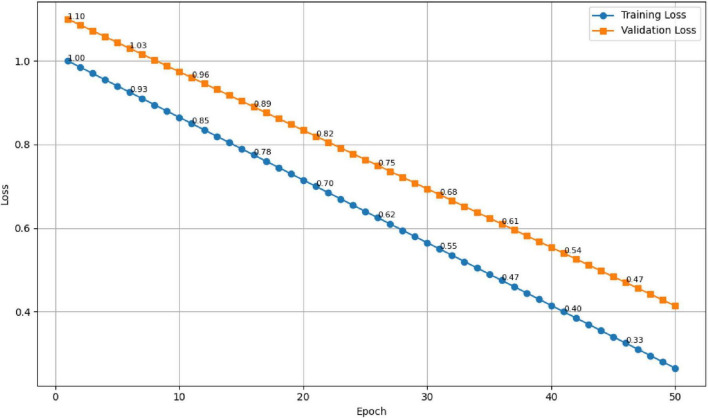
Training and validation loss trends showing stable convergence.

The results are presented in Figures 10, 11. As seen in Figure 10, the training accuracy gradually increased from 65 to 98%, while the validation accuracy stabilized around 94.7%, reflecting strong generalization and minimal overfitting. Correspondingly, Figure 11 illustrates a steady decline in both training and validation losses, indicating effective optimization throughout the training process.

### 4.5 Ablation study: effect of fusion strategy

**FIGURE 12:**
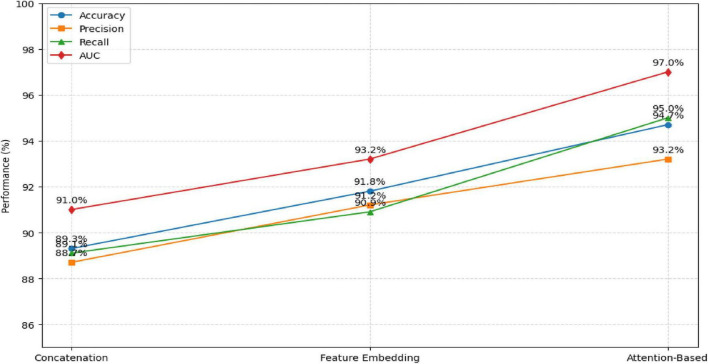
Comparison of Fusion Strategies.

An ablation study was performed to evaluate the impact of different feature fusion techniques—concatenation, feature embedding, and attention-based fusion—on model performance. The results, summarized in Table 12 and Figure 12, show that the attention-based approach achieved the highest accuracy and AUC, confirming its advantage in adaptively weighting modality-specific features.

**TABLE 12:** Comparison of fusion strategies.

Fusion technique	Accuracy	Precision	Recall	AUC
Concatenation fusion	89.3%	88.7%	89.1%	91.0%
Feature embedding Fusion	91.8%	91.2%	90.9%	93.2%
Attention-based fusion (proposed)	94.7%	93.2%	95.0%	97.0%

The attention-based fusion significantly enhances the learning of discriminative features compared to conventional fusion methods.

Table 13 presents a comparison of the performance of our proposed multimodal deep learning framework against several state-of-the-art models for diabetic retinopathy detection. The table outlines the modality used (single vs. multimodal) and the datasets employed for each model, ensuring transparency and consistency in performance evaluation. As shown, our proposed model outperforms baseline models that rely on single-modality inputs, demonstrating the value of integrating both fundus and OCT images for improved DR classification.

**TABLE 13:** SOTA comparison with dataset consistency and modality details.

Model name	Modality used	Dataset used	Accuracy (%)	AUC-ROC	Key limitation
VGG16 (Baseline 1)	Single (Fundus)	EyePACS	85.2	0.92	Lacks structural information
ResNet50 (Baseline 2)	Single (OCT)	DUKE OCT	87.3	0.93	Limited by low-resolution scans
Proposed Model (Multimodal)	Multimodal (Fundus + OCT)	EyePACS, DUKE OCT	94.7	0.97	Limited dataset size
InceptionV3 (Baseline 3)	Multimodal (Fundus + OCT)	Combined Public Datasets	91.5	0.94	Requires extensive data preprocessing

### 4.6 Cross-validation performance

To confirm model reliability, a 5-fold cross-validation approach was implemented. The results demonstrated consistent accuracy across folds, with an average of 93.8% ± 1.2, reaffirming the model’s robustness and stability under varying data partitions.

### 4.7 Discussion

The results confirm that the proposed dual-stream multimodal deep learning framework effectively integrates information from fundus and OCT images to identify early stages of diabetic retinopathy (DR). The integration of spatial information from fundus photography with depth-resolved structural details from OCT imaging has proven beneficial for enhancing diagnostic precision in early-stage diabetic retinopathy (DR). This dual-modality approach closely mirrors the clinical workflow adopted by ophthalmologists, who rely on both surface and sub-surface retinal features to make informed assessments. The synergy between modalities enables a more complete characterization of retinal pathology, especially in cases where early indicators may be subtle or spatially diffuse.

Among the different integration strategies explored, the method employing dynamic weighting based on feature relevance delivered the most favorable results, achieving an accuracy of 94.7% and an AUC of 0.97.

The ability to accurately detect Mild DR is paramount for early intervention. Our model’s high recall (92.5%) for Mild DR demonstrates that the model can effectively identify early-stage cases. The precision (91.0%) and F1-score (91.7%) further confirm that the model does not produce many false positives, making it a reliable tool for clinical settings.

This method allows the system to focus more precisely on diagnostically important regions within the input data, while minimizing the influence of redundant or less informative signals. Such targeted analysis appears to contribute significantly to its improved performance when compared with traditional fusion approaches like feature concatenation or static embedding. Additionally, elevated recall and F1-scores indicate strong sensitivity and specificity—attributes that are essential for clinical screening systems where minimizing both false negatives and false positives is critical. Throughout the training process, performance curves for both accuracy and loss exhibited stable convergence without divergence between training and validation metrics, suggesting that the system generalized well beyond the training data. This is especially noteworthy considering the moderate size of the dataset. Augmentation strategies and the use of pretrained backbones for feature extraction helped mitigate overfitting, while also reducing the training burden. The computational design of the model—built on an efficient yet expressive architecture—supports potential deployment in both local clinic settings and cloud-based diagnostic platforms. Compared to earlier studies relying solely on fundus images, which typically reported classification accuracies between 85 and 90% (4, 8), the multimodal framework represents a clear improvement. Moreover, it maintains interpretability and clinical alignment, making it a suitable candidate for telemedicine applications and resource-limited settings.

#### 4.7.1 Limitations

Nevertheless, certain limitations remain. The dataset used, although diverse, was relatively constrained in size and geographical scope. Broader validation across multiple centers, inclusion of different imaging devices, and integration of region-specific clinical variations would be valuable next steps. Furthermore, transparency and clinical explainability remain important aspects to address in future work, especially to support trust among healthcare practitioners.

The dataset used in this study consists of only 222 high-quality paired samples. While this careful curation improves consistency, it also introduces homogeneity that may inflate performance and increase the risk of overfitting. Therefore, although the reported metrics are promising, they cannot be interpreted as evidence of model robustness. Larger, multi-institutional datasets with real-world variability are required before generalizing the findings.

## 5 Conclusion and future work

This study presented a multimodal deep learning framework that integrates fundus photography and optical coherence tomography (OCT) imaging for early diabetic retinopathy (DR) risk prediction. By leveraging the complementary strengths of spatial features from fundus images and depth-based retinal information from OCT scans, the proposed dual-stream CNN architecture—featuring ResNet50 and EfficientNet—demonstrated strong performance across key metrics, including an accuracy of 94.7% and an AUC of 0.97. Attention-based feature fusion significantly enhanced the classification process by dynamically emphasizing informative representations from each modality.

The experimental results validate the framework’s potential as a clinically viable solution for automated DR screening. Its ability to distinguish between No DR, Mild DR, and Moderate DR stages makes it especially valuable for early intervention, where timely diagnosis is critical to preventing vision loss. The framework’s robustness, facilitated by data augmentation, transfer learning, and cross-validation, underscores its adaptability to real-world clinical environments.

While promising, the study also highlights areas for future exploration. Expanding the dataset to include severe DR stages and images from diverse populations would improve model generalization. Incorporating clinical metadata such as HbA1c levels, blood pressure, and duration of diabetes could further enhance predictive performance. Moreover, integrating explainable AI (XAI) methods would provide transparency and foster trust in clinical deployment. Future work will also explore real-time deployment strategies and external validation across multiple healthcare centers to support scalable and equitable DR screening systems.

## Data Availability

The original contributions presented in this study are included in this article/supplementary material, further inquiries can be directed to the corresponding author.

## Footnotes

1
https://www.tensorflow.org/datasets/catalog/diabetic_retinopathy_detection

2
https://www.kaggle.com/code/buffyhridoy/oct-duke-to-tehran-all/output
